# Botulinum Toxin Type A Exerts Direct Trans-Synaptic Action at Bilateral Spinal Nociceptive Circuits

**DOI:** 10.3390/toxins17030140

**Published:** 2025-03-14

**Authors:** Dalia Nemanić, Petra Šoštarić, Patrik Meglić, Ivica Matak, Lidija Bach-Rojecky

**Affiliations:** 1 Department of Pharmacology, University of Zagreb Faculty of Pharmacy and Biochemistry, A. Kovačića 1, 10 000 Zagreb, Croatia; dalia.nemanic@pharma.unizg.hr; 2 Laboratory of Molecular Neuropharmacology, Department of Pharmacology, Croatian Institute of Brain Research, University of Zagreb School of Medicine, Šalata 11, 10 000 Zagreb, Croatia; petra.sostaric.muzic@ki.se (P.Š.); patrik.meglic@mef.hr (P.M.); 3 Department of Neuroscience, Karolinska Institutet, Solnavagen 9–kvarter B4, 17165 Solna, Sweden

**Keywords:** botulinum toxin type A, trans-synaptic transport, carrageenan-induced bilateral inflammatory pain, neutralizing antitoxin, c-Fos immunohistochemistry, synaptosomal-associated protein 25

## Abstract

Botulinum toxin type A (BoNT-A) induces a bilateral analgesic effect following unilateral injection in rodent bilateral or mirror pain models. This occurs either by indirect plasticity-related actions, or by the toxin’s direct central action in bilateral spinal circuits. Herein, we aimed to resolve this question by assessing the role of trans-synaptic toxin traffic in a bilateral inflammatory pain model. The analgesic effect of the toxin was examined in rats pre-treated with unilateral intraplantar BoNT-A (7 U/kg) and subsequently challenged with bilateral carrageenan-evoked hind-paw inflammation (2%, 50 µL/paw, 6 days post BoNT-A). Specific neutralizing antitoxin injected into the lumbar intrathecal space (2 IU, 24 h post BoNT-A), aimed at preventing the spinal trans-synaptic traffic of BoNT-A, abolished its bilateral analgesic effect. The toxin trans-synaptic effect was associated with reduced c-Fos neuronal activation and BoNT-A-mediated cleavage of synaptosomal-associated protein 25 (SNAP-25) in the bilateral dorsal horn. Here, we showed that, in bilaterally occurring pain, BoNT-A exerts a direct contralateral analgesic action extending beyond the level of the dorsal root ganglion sensory neuron that directly links the hindlimb injection site to the primary sensory region. This points to the crucial role of the toxin’s central trans-synaptic traffic, and its direct action at propriospinal nociceptive circuits in its pain-relieving efficacy.

## 1. Introduction

Botulinum neurotoxin type A (BoNT-A) prevents synaptic transmitter release by the enzymatic cleavage of the synaptosomal-associated protein of 25 kDa (SNAP-25), one of the three synaptic proteins that form the heterotrimeric soluble N-ethylmaleimide-sensitive factor-attachment protein receptor (SNARE) complex involved in the synaptic vesicle fusion with the presynaptic membrane [1]. This specific action at peripheral nerve endings (from motoneurons or postganglionic autonomic neurons) has been the rationale for BoNT-A application in various disorders characterized by skeletal muscle or autonomic (parasympathetic or sympathetic) nervous system hyperactivity [2,3]. In addition, peripherally injected BoNT-A exerts beneficial actions in different chronic pain states, like chronic migraine [4,5]. Research on BoNT-A’s analgesic properties remains a focus of the broader scientific community due to its lasting efficacy in treatment-resistant chronic pain (level A evidence for trigeminal, post-herpetic, and post traumatic neuralgia, level B evidence for diabetic neuropathy, plantar fasciitis, piriformis syndrome, pain associated with total knee arthroplasty, male pelvic pain syndrome, chronic low back pain, and neuropathic pain secondary to traumatic spinal cord injury) [6]. Based on in vitro data and ex vivo studies suggesting that the toxin peripheral injection is associated with reduced peripheral neurotransmitter release and nerve terminal ion channel activity, it was believed that its action was limited to the site of application in the periphery [7]. On the other hand, several lines of observation confirmed that, at the level of consciously behaving experimental animals, the toxin exhibits a central analgesic action causally related to its retrograde axonal transport from the periphery to the central nervous system (CNS) [4,8,9]. Among the most enigmatic properties that moved the focus away from peripheral neuronal terminals is BoNT-A’s bilateral analgesic action after unilateral injection, observed at low non-systemic doses in different bilateral pain models [8]. Consistent results from in vivo experiments suggested a central antinociceptive action associated with some forms of neural plasticity at the level of bilateral spinal cord circuits [4,8,10,11].

Breakthrough experiments done by Antonucci et al. in 2008 [12] were the first to suggest BoNT-A transcytosis within the CNS (i.e., in the rat visual system). Contrary to Cai et al. [13], Caleo et al. in 2018 [14] and Matak in 2020 [15] demonstrated BoNT-A cell-to-cell transport within the rat motor system in the brainstem and spinal cord, respectively. Our recent study demonstrated that BoNT-A undergoes trans-synaptic transport within the brainstem sensory nociceptive trigeminal nucleus caudalis after application into the rat vibrissal pad [9]. Since SNAP-25 is the only known BoNT-A pharmacological target, the truncated SNAP-25 fragment produced by the BoNT-A-mediated specific cleavage of nine C-terminal amino acid residues (SNAP-25 (1-197) or cl-SNAP-25) was utilized as a marker of the toxin’s light chain presence at the distinct sites in biologically active form.

Previously, cl-SNAP-25 was detected in the medullary dorsal horn of the trigeminal nucleus caudalis [16] and spinal cord dorsal horn in different experiments, either ipsilaterally, i.e., on the side of toxin’s application [17,18], or bilaterally [19]. However, the exact localization of the observed contralateral dorsal horn signal was not investigated. One possibility is that the signal originates from the central axons of the primary afferent nerves that terminate contralaterally [20]. Another possibility is that BoNT-A exerts trans-synaptic transport within the spinal cord dorsal horn, as was already seen in the central visual system, brainstem and spinal motor nuclei, and the trigeminal nucleus caudalis.

Thus, in the present study, we analyzed the possible trans-synaptic origin of BoNT-A’s bilateral antinociceptive efficacy within the lumbar spinal cord by examining the toxin’s behavioral antinociception and its effects on bilateral dorsal horn neuronal activation. The toxin’s trans-synaptic traffic was prevented by specific intrathecally applied neutralizing antitoxin, while its direct central antinociceptive actions were further characterized by the quantification of c-Fos neuronal activation in the dorsal horn, as well as the toxin’s enzymatic activity at its well-known synaptic target cl-SNAP-25.

## 2. Results

### 2.1. The Role of BoNT-A Transcytosis in the Lumbar Spinal Cord

As a main study goal (Experiment 1) we aimed to investigate if BoNT-A’s bilateral antinociceptive action involves its trans-synaptic transport at the level of the first sensory synapse in spinal cord dorsal horn. We induced bilateral inflammatory mechanical hyperalgesia with carrageenan and explored the antinociceptive effect of unilateral BoNT-A pre-treatment on both sides. Furthermore, to investigate the role of BoNT-A trans-synaptic traffic from the primary afferent nerve endings in its antinociceptive effect, we employed neutralizing antitoxin (2 IU, i.t.), 24 h after the peripherally delivered BoNT-A (7 U/kg, i.pl.).

#### 2.1.1. BoNT-A Reduces Bilateral Paw Pressure Nociception, Dependent on Its Transcytosis, with No Effect on Motor Performance

**Figure 1:**
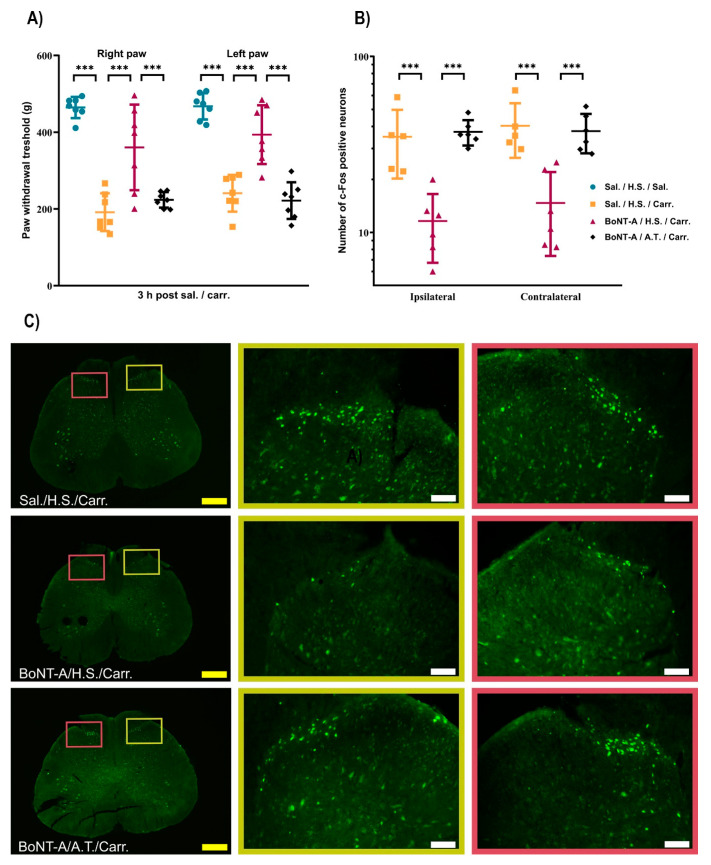
Bilateral antinociceptive effect of BoNT-A in the experimental inflammatory pain induced by carrageenan is dependent on spinal toxin trans-synaptic traffic. The effect of spinal application of antitoxin to BoNT-A (2 IU, applied i.t. 24 h post toxin) on (**A**) antinociceptive effect of BoNT-A in paw pressure test and (**B**) BoNT-A effect on bilateral c-Fos neural expression (examined 3 h post carrageenan). (**C**) Representative examples of fluorescently-labeled c-Fos (green punctate immunoreactivity) in the ipsilateral (right, the side of BoNT-A pre-treatment, yellow frame) and contralateral (red frame) sides of dorsal horns at L4/L5 spinal cord sections. Mechanical sensitivity (expressed in grams, g) was measured with a paw pressure withdrawal test 3 h post carrageenan (2%) and 6 days after unilateral BoNT-A (7 U/kg) administration subcutaneously under the right hind-paw plantar skin (number of animals per group = 7). Average number of c-Fos positive neurons for each animal was assessed 3 h after carrageenan application (mean of four sections per animal, number of animals per group = 5–6). Behavioral results (**A**) are expressed as mean ± SD, while immunohistochemical quantification (under **B**) is represented on a logarithmic scale as median with interquartile range; untransformed (**A**) or square root-transformed data (**B**) were analyzed by a linear mixed model followed by a two-stage linear step-up procedure of Benjamini, Krieger, and Yekutieli to correct for multiple comparisons by controlling the false discovery rate (<0.05); *** = *p* < 0.001. Scale bar in yellow represents 500 µm (4× magnification) or in white represents 100 µm (20× magnification). Abbreviations: Sal. = saline; H.S. = horse serum; A.T. = antitoxin to BoNT-A; Carr. = carrageenan.

Three hours after bilateral carrageenan (2%, i.pl.) application, the rats developed hind-paw inflammation associated with bilateral mechanical hyperalgesia. Peripheral BoNT-A pre-treatment significantly reduced the mechanical hypersensitivity elicited by carrageenan application. Although the BoNT-A was injected unilaterally, this effect was significant on both ipsilateral and contralateral paws. In the experimental group of rats treated with the neutralizing antitoxin, BoNT-A’s beneficial effect on either side was prevented (Figure 1A), suggesting the involvement of the toxin’s central trans-synaptic traffic in its analgesic actions. Basal nociceptive threshold values (measured on day 0) did not differ between experimental groups (Supplementary Material, Table S1).

To exclude a systemic effect of 7 U/kg BoNT-A (due to the toxin’s possible systemic distribution and/or local toxin action) on motor performance, we measured the rat weight and motor performance prior to BoNT-A injection and then on day 6 before the bilateral carrageenan application. We excluded the occurrence of systemic toxin action and possible influence of BoNT-A-evoked muscle weakness on mechanical hyperalgesia measurements, since the results of the rota-rod test did not differ between BoNT-A treated animals vs. controls (Table 1).

#### 2.1.2. The BoNT-A Trans-Synaptic Effects Are Associated with the Reduced Nociceptive Neuronal Activation in the Bilateral Dorsal Horn

To further test the spinal effect of BoNT-A action on inflammatory hyperalgesia, we performed an immunohistochemical analysis of c-Fos, a marker of the pain-evoked neuronal activation of nociception-specific and wide dynamic range neurons within the dorsal horn. While carrageenan induced a significant bilateral elevation of c-Fos expression in the spinal cord L4/L5 segments, pre-treatment with BoNT-A significantly decreased the c-Fos expression on both, the toxin-injected and non-injected side. In line with behavioral results, neutralizing antitoxin to BoNT-A abolished the mentioned toxin-mediated preventive effect on neural activation at both dorsal horns (Figure 1B,C).

#### 2.1.3. The BoNT-A Undergoes Trans-Synaptic Traffic in the Dorsal Horns Following Unilateral Hind-Paw Injection

**Figure 2:**
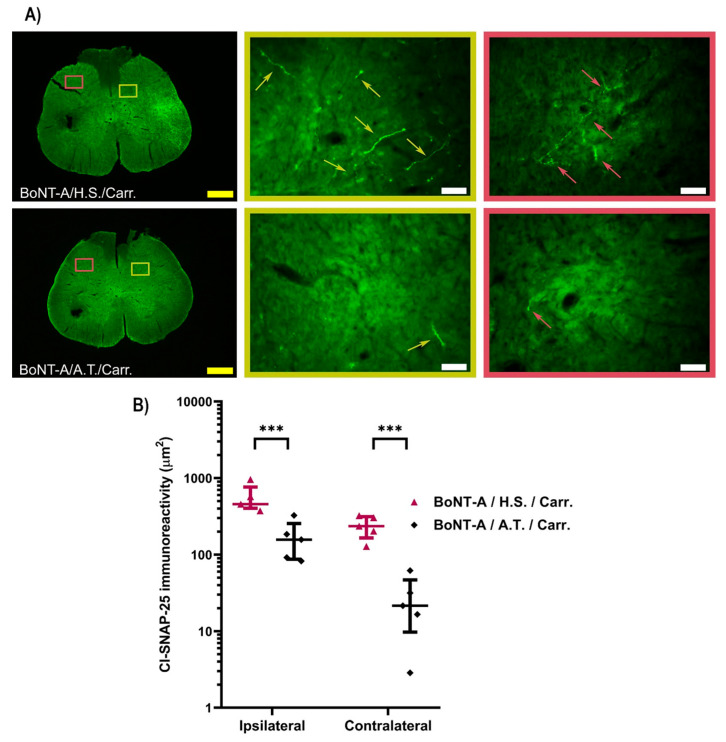
The BoNT-A enzymatic activity in the bilateral dorsal horns of the spinal cord depends on the toxin’s trans-synaptic transport. (**A**) Representative images of BoNT-A proteolytic activity (green immunostaining pointed by arrows) in the ipsilateral (yellow frame and arrows) and contralateral (red frame and arrows) sides of the spinal cord dorsal horns. Scale bar in yellow represents 500 µm (4× magnification) and in white represents 100 µm (20× magnification). (**B**) Antitoxin to BoNT-A applied intrathecally reduced the cl-SNAP-25 immunoreactivity in the ipsilateral and contralateral side spinal cord dorsal horn. The data are representative of five animals per group. Average surface of immunoreactivities of cl-SNAP-25 for each animal was calculated from three spinal cord sections per segment (L3, L4, and L5/6). (N(animals/group) = 5). Results are expressed on a logarithmic scale as median with interquartile range, with the statistical analysis performed on square root-transformed data and further analyzed by a linear mixed model followed by a two-stage linear step-up procedure of Benjamini, Krieger, and Yekutieli to correct for multiple comparisons by controlling the false discovery rate (<0.05); *** = *p* < 0.001. Abbreviations: H.S. = horse serum; A.T. = antitoxin to BoNT-A; Carr. = carrageenan.

To test BoNT-A’s distribution at distant sites from the application, i.e., in the spinal cord, here we employed the occurrence of the BoNT-A-truncated SNAP-25 fragment (cl-SNAP-25) as a marker of BoNT-A proteolytic activity. In BoNT-A treated rats, cl-SNAP-25 immunoreactivity was detected in bilateral dorsal horns (Figure 2) and ventral horns at the L3-L5/6 level of the spinal cord (Figure S1). The contralateral immunoreactivities were lower compared to the ipsilateral side.

Antitoxin applied intrathecally at the L3/L4 level one day after BoNT-A i.pl. application significantly reduced the occurrence of cl-SNAP-25 immunoreactivity on both sides (Figure 2A,B).

#### 2.1.4. The Preventive Effects of i.t.-Applied Antitoxin Are Not Due to Systemic Distribution After Lumbar i.t. Application

In Experiment 2, we examined the slight possibility that the antitoxin may reach systemic circulation after the absorption and distribution from the i.t. application site, and that this peripherally distributed fraction of the antitoxin may be responsible for the observed prevention of BoNT-A central enzymatic action on SNAP-25 cleavage. To evaluate any peripheral antitoxin effect, we compared the effect of systemically distributed antitoxin (injected i.m.) to the effect of i.t. antitoxin on the occurrence of cl-SNAP-25 in the ipsilateral ventral horn. The peripherally injected antitoxin was applied into the left vastus lateralis muscle on the side contrary to the i.pl. BoNT-A application. The cl-SNAP-25 immunoreactivity was quantified in the ipsilateral and contralateral ventral horns (L5/6 segments) of the spinal cord. The ventral horn was chosen because of the higher occurrence of the cl-SNAP-25 in comparison to the dorsal horns. The results showed that, in comparison to i.t.-injected antitoxin, the intramuscularly applied neutralizing antitoxin did not change the occurrence of cl-SNAP-25-positive fibers on either side of the ventral horns when compared to the antitoxin non-treated animals (Figure S2). This experiment excludes the importance of other sites of i.t. antitoxin action apart from the spinal cord extracellular interstitial fluid contained within the blood–brain barrier, and the possibility of antitoxin systemic distribution and peripheral nerve endings as important sites of its neutralizing activity.

## 3. Discussion

In the present work, we showed that BoNT-A’s antinociceptive effect on bilateral pain involves the toxin’s trans-synaptic transport at the level of the spinal cord lumbar segment associated with the sensory innervation of the pain-affected area. The centrally applied neutralizing antitoxin specific for BoNT-A counteracted (a) the BoNT-A-mediated beneficial effect on mechanical hyperalgesia, (b) the BoNT-A-mediated reduction of c-Fos nociceptive neuronal activation, and (c) cl-SNAP-25 occurrence on the ipsilateral side, but also on the contralateral side as well. These results are in line with our recent experiments which demonstrated BoNT-A’s trans-synaptic transport within the trigeminal nucleus caudalis after the toxin’s injection into the rat vibrissal pad [9].

Here, we employed a model of bilateral inflammatory pain induced by intraplantar carrageenan injections, as previously described by Favre-Guilmard et al. in 2017 [21]. They investigated the time-dependent effects of unilateral abobotulinumtoxinA (aboBoNT-A, 20, 30 U/kg) on carrageenan-induced bilateral inflammatory pain in rats. The results showed that aboBoNT-A injected 3 days, but not 1 day, before carrageenan prevented hyperalgesia in both the treated and untreated inflamed paws. The authors concluded that the bilateral activation of sensory neurons is a prerequisite for BoNT-A’s bilateral effect but indicated that “future studies evaluating diffusion and migration of the toxin away from the injection site can shed light on mechanisms of this phenomenon” [21]. Previous findings related to unilateral carrageenan-evoked pain suggested that contralateral toxin injection does not exert antinociceptive action on the uninjected side [10]. This is in line with the possibility that contralateral toxin antinociceptive actions depend on the ongoing nociceptive input from the contralateral side.

Nowadays, it has been widely confirmed that the toxin’s retrograde axonal transport to the central nervous system from the periphery is a prerequisite for its effect on central pain processing. Previously, based on colocalization experiments, we discovered that BoNT-A’s axonal transport to the CNS is mediated by TRPV1-expressing sensory neurons [22]. However, this does not necessarily exclude other neuronal pathways since the detection of BoNT/A in central neurons might be limited by the immunodetection method that does not necessarily distinguish low numbers of cleaved SNAP-25 molecules (as a marker of toxin’s proteolytic activity) that still might interfere with synaptic transmitter release.

The cleaved SNAP-25 is commonly detectable as individual fibers by the present immunohistochemical methods. The detection of continuous SNAP-25 immunoreactivity in fibers might suggest the presence of more BoNT-A molecules that potentially distribute along the axons. Presently, it is not clear which fraction of sensory synapses (involved in the toxin’s antinociceptive activity) are affected by BoNT-A. However, it is estimated that only a few molecules of BoNT-A per synapse might inhibit the synaptic vesicle release. In addition, the toxin’s effect on synaptic release prevention may be augmented by the long-term presence of cleaved SNAP-25 in the synapse and its ability to form inactive SNARE complexes (a single inactive SNARE heterotrimer may inactivate the entire SNARE supercomplex at the active release site) [23]. Therefore, detecting a few cleaved SNAP-25 molecules that exert synaptic blockades might be challenging by the present detection method. Interestingly, in the present study, after the toxin’s application into the hind-paw pad, we observed a significantly higher magnitude of cl-SNAP-25 expression in the ventral horns in comparison to the dorsal horns, which is in line with observations by other authors [24].

As mentioned, by employing the cleaved SNAP-25 as a specific marker of the toxin’s proteolytic activity, several groups demonstrated the presence of cl-SNAP-25 immunofluorescent labeling in the spinal cord dorsal horns ipsilaterally [14,15,16] and bilaterally [17,20] after BoNT-A peripheral application. In line with a bilateral cl-SNAP-25 signal in the trigeminal nucleus caudalis after the toxin’s unilateral injection into the rat vibrissal pad [9], here, we also observed a cl-SNAP-25 signal on both examined sides of the spinal cord.

Based on a well-known mechanism of BoNT-A action within the peripheral cholinergic synapses, it was widely hypothesized that it reduces pain by cleaving SNAP-25 and consequently by preventing the exocytosis of excitatory neurotransmitters/inflammatory mediators from the local primary afferent nerve endings adjacent to the injection site, and indirect interference with the processes of central sensitization [7]. However, as mentioned, this assumption could not explain the bilateral analgesic actions of BoNT-A reported in many different polyneuropathic [4,25] and mirror-image pain models [8,18]. There was also a possibility of indirect action, wherein the toxin remains localized on the ipsilateral-side dorsal horn, but nevertheless indirectly affects contralateral nociceptive processing through the central plasticity-associated neuronal changes or by preventing the circulating neuronal mediators or mediators secreted by activated glia, provided that these factors are involved in contralateral-side pain. Thus, BoNT-A could directly and/or indirectly interact with various neural systems and glia cells within the CNS [4,25]. Even if the BoNT-A antinociceptive actions in inflammatory pain are somewhat different compared to mirror-image pain, a previous study from another group [21], and our recent independent study [9], provide support for a direct bilateral effect (and not an indirect contralateral effect derived from a direct ipsilateral effect) of the toxin. It was intriguing to speculate that the toxin might directly affect other cells (second-order sensory neurons, interneurons, glia cells, and descending pain inhibitory pathways), with the presumption that it leaves the primary afferent neurons in its biologically active form. Indeed, an in vitro experiment by Restani et al. [26] suggested that retrograde trafficking of BoNT-A occurs in vesicles that escape acidification, allowing the transcytosis of a full-length toxin. Experiments performed on animals that investigated the toxin’s transcytosis employed a classical pharmacological approach with the BoNT-A-specific neutralizing antibody applied centrally (at the level of the first synapse) [14,15]. Given that antitoxin can only neutralize extracellular toxin molecules [27], current findings suggest that a portion of BoNT-A exits the central terminals of the primary afferent neurons and enters second-order central synapses. Similarly, the neutralizing antitoxin prevented BoNT-A’s central effects that were responsible for its motor actions in hyperactive skeletal muscles of the hind limb [15].

The most recent experiments performed in our lab [9] for the first time showed that neutralizing antitoxin to BoNT-A significantly reduced not only its contralateral antinociceptive effect in the craniofacial region, but also cl-SNAP-25 expression within the sensory nervous system on both the toxin-injected and non-injected sides of the rat face. However, this study was performed in the trigeminal nerve-innervated craniofacial region, which did not allow the generalization of reported findings to other extracranially innervated regions and limbs. Thus, in the present study, this was the main motive to test whether similar results could be obtained in the sciatic nerve-innervated area.

Furthermore, in the present study, we wanted to exclude the possibility that any fraction of the antitoxin (that possibly diffused out of the site of its intrathecal application into circulation) may somehow interact with BoNT-A traffic to the spinal cord outside of the CNS (i.e., not by central BoNT-A neutralization). To test this possibility, we injected three additional animals with the antitoxin applied i.m. (into the vastus lateralis muscle) to facilitate its systemic delivery (similarly to the i.m. antitoxin used clinically to prevent botulism) and compared the effect of i.t. and i.m. antitoxin on the occurrence of cl-SNAP-25 in the ventral horn (Experiment 2). Although the experiment was performed on just three animals per examined group, the obtained results based on analyses of multiple spinal cord sections suggest a lack of any significant systemic effect of the antitoxin, excluding other important sites of BoNT-A neutralization (apart from central intraspinal extracellular interstitial fluid) that may reduce its central enzymatic actions (Figure S2). It also confirms, in line with the well-established time-course of BoNT-A internalization into the nerve terminal [1,4], that the peripherally injected toxin destined for axonal transport to the CNS enters quickly into the peripheral nerve terminal and then remains inaccessible to subsequently applied systemic antitoxin. In line with the similarity with the trafficking of neurotrophins or pathogen-derived molecules such as tetanus toxin (that involves axonal transport via peripheral neurons, followed by exocytosis and specific internalization to second-order central synapses), the neutralization of the toxin activity at the level of the CNS may occur only at the point when the holotoxin molecules (containing both heavy and light chain) are re-exposed by exocytosis from motoneurons or primary afferents into the extracellular inter-synaptic milieu, and before its subsequent heavy chain-mediated specific membrane binding and entrance at second-order synapses [9,14,15,25].

**Figure 3:**
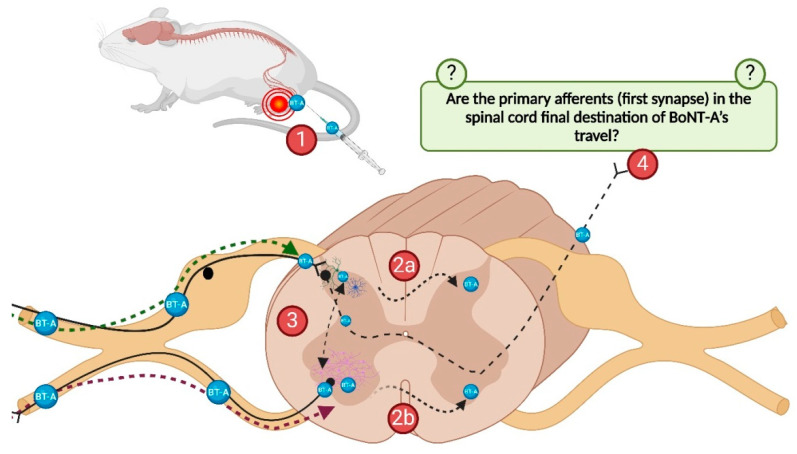
Possible destinations of centrally transported BoNT-A after its application into the hind-paw. Peripheral BoNT-A application and retrograde or transganglionic transport to the CNS via motor and sensory neurons (1). Putative trans-synaptic transport by contralaterally projecting primary afferent central terminals (2a) or via commissural neurons between dorsal horns (2a) and ventral horns (2b), or ipsilateral (inter)neurons (3). Possible BoNT-A transport to the higher-order central nuclei via long-range projections (e.g., spinothalamic or spinoreticular pathway) (4). Created with BioRender.com.

The neural pathways employed by BoNT-A to leave the primary afferent neurons and reach contralateral central synaptic targets remain unknown. One of the hypothetical trafficking mechanisms (Figure 3) could rely on the BoNT-A transfer via commissural interneurons to the contralateral side of the spinal cord [20,28]. The contralateral trans-synaptic traffic, as well as the partial persistence of cl-SNAP-25 on the contralateral side despite antitoxin administration, could also be attributed to central primary afferent terminals that project directly to the contralateral dorsal horn. This possibility is supported by a recent report [20] that up to 27% of Lamina I neurons, including projection neurons, receive direct input from contralateral Aδ- and C-fibers (Figure 3). It was shown that these afferents supplying lumbar Lamina I neurons with contralateral input are under inhibitory control. The disinhibition of these pathways significantly amplifies nociceptive drive, which explains the phenomena of contralateral hypersensitivity and mirror-image pain [20]. Consequently, this supports the theory that the bilateral antinociceptive effect of unilaterally administered BoNT-A is mediated by central mechanisms.

Our findings in the ventral horns (Figures S1 and S2) align with previous findings that intrathecally administered antitoxin inhibited SNAP-25 cleavage in the spinal cord ventral horn following the intramuscular and intraneural application of BoNT-A into the sciatic nerve [15]. Another important question that awaits further characterization is determining the exact central spinal trans-synaptic targets that may contribute to the toxin’s analgesic efficacy. Along with established action at TRPV1-expressing central afferent terminals, these trans-synaptic targets are most likely central excitatory synapses that may belong to various propriospinal interneurons and/or descending excitatory inputs. Based on previous data that demonstrated the prevention of BoNT-A antinociceptive action by short-acting pharmacological antagonists, these neuronal targets of BoNT-A (along with central afferent terminals) may also interact indirectly with other pain-modulating systems and receptors, such as the endogenous opioid system (via µ-opioid receptor) and inhibitory GABA-ergic neurons (via GABA-A receptor) [4,8,18].

The present study has some limitations. First, the experiments were performed on male adult rats (did not include female rats), and tested the effect of a single BoNT-A dose at a single time-point. Thus, BoNT-A’s possible dose- and time-dependent effects on pain and cl-SNAP-25 accumulation need additional investigations. Furthermore, the pathway of BoNT-A traffic to the contralateral central synapses (either by contralaterally projecting central afferent terminals or by commissural interneurons) cannot be distinguished by the present study and deserves further experimentation. Also, the identification of the exact type of second-order dorsal horn synapses directly affected by BoNT-A awaits a more complex approach involving genetic characterizations of defined dorsal horn synapses/neuronal populations.

## 4. Conclusions

Presently, we demonstrate that, in bilaterally occurring pain, the BoNT-A exerts a direct contralateral analgesic action that might rely on the toxin’s central trans-synaptic traffic and its direct action at contralateral propriospinal nociceptive circuits. Altogether, these data suggest a complex interaction of the toxin with spinal nociceptive circuits as the basis of its antinociceptive action. A more complete understanding of these interactions might provide an important research tool for the interpretation of the BoNT-A clinical analgesic action, as well as to elucidate clinically relevant pain mechanisms, in general.

## 5. Materials and Methods

### 5.1. Animals

Adult male Wistar rats (4–5 months old) weighing 450–550 g were used in all of the experiments. Three rats per home cage were housed at the animal facility of the Department of Pharmacology, University of Zagreb School of Medicine, Croatia, in a standard temperature (21–23 °C) and relative humidity (40–70%) regulated environment, under a 12 h light/dark cycle, with food and water ad libitum. All of the experimental procedures were approved by the Ethical Committee of the University of Zagreb School of Medicine and by the Croatian Ministry of Agriculture (permit: 386/2023), and performed in agreement with the European Union Directive 2010/63/EU and ARRIVE guidelines 2.0: Updated guidelines for reporting animal research [29].

### 5.2. Drug Administration and Experimental Protocol

**Figure 4:**
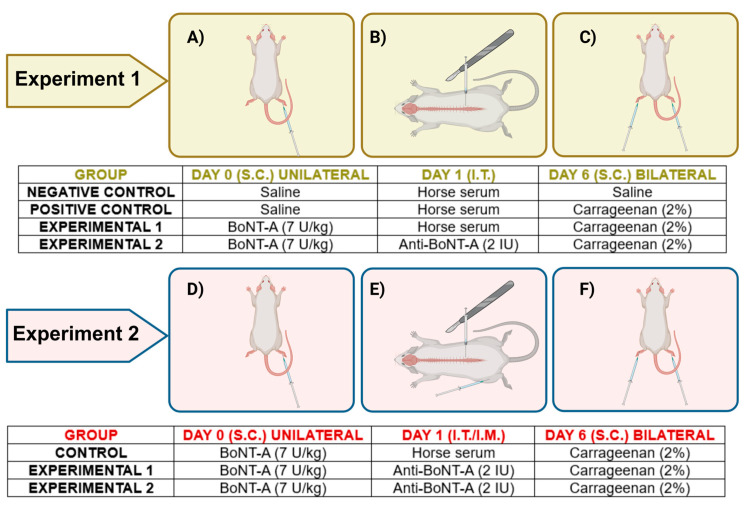
Experimental protocols. Experiment 1: Animals (28) were randomized and assigned into four experimental groups. The first group (negative control) was (**A**) injected with saline (unilateral, i.pl.) on day zero, (**B**) with the horse serum (2 IU/10 µL, per rat, i.t.) the following day, and (**C**) with saline into both paws on day 6. The second group (positive control) was (**A**) injected with saline (unilateral, i.pl.) on day zero, (**B**) with the horse serum (2 IU/10 µL, per rat, i.t.) the following day, and (**C**) with carrageenan into both paws to induce the inflammation on day 6. The third and fourth groups (experimental groups 1 and 2, respectively) were (**A**) injected with BoNT-A (unilateral, i.pl.) on day zero, (**B**) with either horse serum or antitoxin for BoNT-A (2 IU/10 µL, per rat, i.t.) the following day, and (**C**) with carrageenan into both paws on day 6. Experiment 2: Animals (9) were randomized and assigned into three experimental groups. The first group (control) was (**D**) injected with BoNT-A (unilateral, i.pl.), (**E**) with the horse serum (2 IU/10 µL, per rat, i.t.) the following day, and (**F**) with carrageenan into both paws to induce the inflammation on day 6. The second and third groups (experimental groups 1 and 2, respectively) were (**D**) injected with BoNT-A (unilateral, i.pl.) on day zero, (**E**) provided i.t. or i.m. with antitoxin for BoNT-A (2 IU/10 µL, per rat) the following day, and (**C**) injected with carrageenan into both paws on day 6. The image was created with BioRender.com. Abbreviations: i.pl. = intraplanar; i.t. = intrathecal; i.m. = intramuscular.

Two independent experiments were performed (Figure 4). The total number of animals used in experiment 1 was 28 (7 in 4 groups) and in experiment 2 was 9 (3 in 3 groups). Animals were randomly assigned to the experimental groups by a person unaware of the treatment. On the last day of experiments, animals were euthanized for tissue harvesting.

#### 5.2.1. Intraplantar Injection

Awake and lightly restrained animals were injected subcutaneously (29-gauge needle) into the plantar surface of the right hind paw (intraplantarly, i.pl.) with 7 U/kg BoNT-A (onabotulinum toxin type A, Botox^®^, Allergan Inc., Irvine, CA, USA) diluted in a 20 µL 0.9% saline vehicle. Each vial of Botox^®^ contains 100 units (U) (~4.8 ng) of purified Clostridium botulinum type A neurotoxin complex. Control groups received, in the same way, 20 µL of 0.9% saline (Figure 4A,D). The dose of BoNT-A was chosen based on the previous experiments from our laboratory.

#### 5.2.2. Intrathecal Injection

The following day (24 h post BoNT-A), animals were anesthetized intraperitoneally with 70/7 mg per kg ketamine/xylazine (Ketamidor^®^ 10%, Richter Pharma AG, Wels, Austria/Xylased Bio^®^ 20 mg/mL, Bioveta, Ivanovice na Hané, Czeck Republic). After shaving the lumbosacral area, a small skin incision was made at the lumbar L4/L5 level of the spinal cord. The animals were injected intrathecally (i.t.) by a 28G × ½’’ 0.5 mL tuberculin syringe, as previously described [15], with 2 IU antitoxin for BoNT-A (lyophilized polyclonal equine IgG-based BoNT-A antitoxin—from the National Institute for Biological Standards and Control, Potters Bar, UK, NIBSC code 14/174, provided by Thea Sesardic, PhD, and Paul Stickings, PhD) or with 20% horse serum (Gibco, ThermoFisher Scientific, Waltham, MA, USA), both diluted in saline to obtain a total volume of 10 µL per rat (Figure 4B,E). A single unit of antitoxin for BoNT-A can neutralize 10,000 mouse LD_50_ doses of BoNT-A. The accuracy of i.t. application was verified by the animal’s tail or hind limb brisk move, and afterwards the skin was sutured. The dose and timing of the antitoxin administration were chosen based on previous studies [14,15] and preliminary experiments performed in our laboratory.

#### 5.2.3. Intramuscular Injection

Additionally, the following day (24-h post BoNT-A) in experiment 2, animals were anesthetized intraperitoneally with 70/7 mg per kg ketamine/xylazine. Antitoxin for BoNT-A (2 IU/10 µL) was applied intramuscularly (i.m.) into the contralateral vastus lateralis (Figure 4E) with a Hamilton syringe needle (0–10 μL Hamilton Microliter #701, Hamilton, Bonaduz, Switzerland).

### 5.3. Carrageenan-Induced Inflammatory Pain Model

In both experiments, animals were lightly anesthetized with gaseous isoflurane (Isofluran-Piramal^®^, Piramal Healhcare, Morpeth, Northumberland, UK) and injected with 2% carrageenan (λ-Carrageenan^®^, Sigma-Aldrich, St. Louis, MO, USA) dissolved in 0.9% NaCl (100 µL) into the plantar surface of both hind paws 6 days after the BoNT-A application (Figure 4C,F). Carrageenan causes a strong inflammatory reaction accompanied by edema and mechanical hyperalgesia, which peaks 3–5 h after administration [21]. The control group of rats received 0.9% saline in the same volume as those treated with carrageenan. The term ipsilateral represents the right paw (BoNT-A application side), while contralateral indicates the left paw, opposite to the BoNT-A application side.

### 5.4. Behavioral Testing

Behavioral experiments were performed in a quiet room between 9 a.m. and 2 p.m. Animals were habituated to the testing environment for 10–15 min. All measurements were performed by experimenters blinded to the animal treatment.

#### 5.4.1. Rota-Rod Test

To test motor performance, rats were trained to maintain balance on a rota-rod device with an 8-cm-diameter rod rotating at a 13 rpm constant rate. The cut-off time (time before falling) was set to 180 s. Animals were tested before BoNT-A application and then after 6 days, before carrageenan application. This test was employed to exclude any influence of BoNT-A on motor performance, which might impact the evoked motor behavioral reaction to mechanical stimuli [30].

#### 5.4.2. Mechanical Hyperalgesia Measurement

Sensitivity to mechanical stimuli was measured on both hind paws using an analgesiometer (Ugo-Basile paw pressure analgesiometer 38500 PAM, Gemonio, Italy), which is a modified version of the paw pressure test originally described by Randall and Selitto in 1957 [31]. Initially, rats were trained for handling by the experimenter for several days before the obtainment of basal nociceptive threshold values (day 0). On day 6, measurements of mechanical hypersensitivity were done before carrageenan, and then at 3 h post carrageenan application. Pressure was applied to the dorsal and ventral sides of both paws, until the paw withdrawal or struggling appeared. Average paw withdrawal threshold expressed in grams was calculated from 3 repeated measurements per paw made in 10 min intervals.

### 5.5. Immunohistochemistry

Upon the completion of behavioral measurement, the animals were deeply anesthetized by ketamine and xylazine (70/7 mg/kg i.p.), and then euthanized by transcardial perfusion with saline (0.9%, 250 mL), followed by fixative consisting of 4% paraformaldehyde in 0.01 M phosphate buffered saline (PBS; pH = 7.4, 250 mL). Lumbar spinal cords were removed and cryoprotected in 15% sucrose-fixative solution overnight, and transferred afterwards in 30% sucrose in PBS. After the tissue sank to the bottom of the container, it was taken out and briefly placed on a paper tissue to remove excess fluid, and later stored at −80 °C. Lumbar spinal cord coronal sections (35 μm) from L3 to L6 were cut on the cryostat (Leica CM 1950, Wetzlar, Germany) and transferred to PBS-filled wells. Prior to cutting, the white matter located ventromedially to the left ventral horn was carefully punctured with a tuberculin syringe needle to mark the spinal cord side contralateral to BoNT-A injection.

#### 5.5.1. C-Fos Immunohistochemistry

Lumbar spinal cord L4/L5 segments of 5–6 rats per each experimental group were cut and prepared for free-floating immunohistochemical analysis. Sections were rinsed in 0.25% Triton X-100 (Sigma-Aldrich, St. Louis, MO, USA) PBS solution (PBS-T) for 3 × 5 min and blocked for 1 h in 10% NGS (normal goat serum, Millipore, Burlington, MA, USA). Afterwards, the sections were incubated with rabbit anti-c-Fos polyclonal antibody (Santa Cruz Biotechnology Inc., Santa Cruz, CA, USA) 1:400 diluted in 1% NGS overnight at room temperature. The next day, the tissue was washed with PBS-T, and incubated with 1:400 goat anti-rabbit Alexa Fluor-488 secondary antibody (Molecular Probes, Invitrogen, Carlsbad, CA, USA) diluted in 1% NGS for 2 h at room temperature in the dark. At the end, slices were rinsed in PBS-T, mounted on glass adhesion slides (Super Frost Plus Gold, Thermo Scientific, Waltham, MA, USA) and protected with anti-fading agent (Fluoroshield with DAPI, Sigma-Aldrich, St. Louis, MO, USA).

The visualization of four randomly selected sections per animal was done with the fluorescent microscope (Olympus BX-51 microscope coupled to DP-70 digital camera, Olympus, Tokyo, Japan) at 4× and 20× magnification. The number of c-Fos-positive neurons in sensory laminas (I and II, and V and VI) of the spinal cord ipsilateral and contralateral dorsal horn was automatically counted in the obtained 20× microphotographs by employing free Fiji Image-J software 2.9.0 (open source under the GNU General Public License, Wayne Rasband and contributors, National Institutes of Health, Bethesda, MD, USA). Figures were processed for brightness and contrast using Adobe Photoshop 2021 (version 22.0.0., Adobe Systems Incorporated, San Jose, CA, USA).

#### 5.5.2. Cleaved SNAP-25 Immunohistochemistry

Sections of the lumbar spinal cord (L3, L4, and L5/L6 segments) of 5 rats per experimental group were used for free-floating immunohistochemical analysis. Immunohistochemical staining was performed according to the manufacturer’s guidelines for goat anti-rabbit Alexa Fluor™ 488 Tyramide SuperBoost™ Kit (Invitrogen by Thermo Fisher Scientific, Eugene, OR, USA). Non-affinity purified rabbit antiserum anti-SNAP-25(1-197) (National Institute for Biological Standards and Control, Potters Bar, UK; kindly provided by Thea Sesardic, PhD) diluted 1:8000 in 1% NGS was incubated overnight at a room temperature. Sections were rinsed and mounted on glass slides with anti-fading agent and left overnight at +4 °C.

Afterwards, three randomly selected sections of L3, L4, or L5/L6 spinal cord segments per animal in Experiment 1 were visualized and photographed at 4×, 10×, and 40× magnification. The average area of cl-SNAP-25 immunoreactivity (in µm^2^) for a segment per animal was quantified in three non-overlapping visual fields of dorsal and ventral horns at both ipsilateral and contralateral sides. The average surface of immunoreactivities of cl-SNAP-25 for each animal was calculated from 3 spinal cord sections per segment (L3, L4, and L5/6). Three non-overlapping visual fields per section were analyzed at 40× magnification images (rectangle dimensions = 436.6 µm by 330.2 µm). Each segment was quantified separately, and the average area was summated (i.e., ipsilateral dorsal horns L3 + L4 + L5/6).

In Experiment 2, three randomly selected sections of the L5/L6 spinal cord segment (ventral horns) per animal were visualized with the Axio Observer 7 fluorescent microscope connected to the Axiocam 305 Color camera with a 0.63× camera adapter (Zeiss, Oberkochen, Germany). Cl-SNAP-25 immunoreactivity (µm^2^) per animal was quantified similarly as described above in three non-overlapping visual fields within ipsilateral and contralateral sides of ventral horns.

In both experiments, images used for quantitative analysis were taken at 40× magnification under constant exposure, while the pixel-threshold area of a single slice was measured with Fiji Image-J software 2.9.0 utilizing the manual threshold range similarly as previously described [15]. Representative microphotographs shown in figures were processed for brightness and contrast using Adobe Photoshop.

### 5.6. Statistical Analysis

Statistical analysis and graph drawing were made using GraphPad Prism 8 (version 8.01, GraphPad Software, Inc., La Jolla, CA, USA). The c-Fos and cl-SNAP-25 data were subjected to square root transformation to normalize the distribution. We obtained Q-Q plots from the model, which showed that there was no deviation from normal distribution. A linear mixed model (LMM) was used, with spinal cord side or paw side as fixed factors and rat identifier as a random intercept. This was followed by a two-stage linear step-up post-hoc of Benjamini, Krieger and Yekutieli to correct for multiple comparisons by controlling the False Discovery Rate (<0.05). Significant values were considered starting at *p* < 0.05 after adjustment.

## Figures and Tables

**Table 1:** The lack of BoNT-A (7 U/kg i.pl.) effect on rat body weight or rota-rod performance.

Treatment/Test (Mean ± SEM)	Sal./H.S./Sal.	Sal./H.S./Carr.	BoNT-A/H.S./Carr.	BoNT-A/A.T./Carr.
Weight (g) pre-BoNT-A	536.57 ± 15.6	536 ± 13.92	515.42 ± 13.35	523.71 ± 13.63
Weight (g) 6 days post-BoNT-A	528 ± 15.49	520 ± 17.59	498 ± 12.22	502 ± 15.69
Rota-rod latency (s) pre-BoNT-A	138.28 ± 14.18	118.14 ± 16.72	124.71 ± 17.73	125.71 ± 16.08
Rota-rod latency (s) 6 days post-BoNT-A	143 ± 10.64	109.14 ± 19.02	114.29 ± 19.41	120 ± 13.49

Legend: Sal. = saline; H.S. = horse serum; A.T. = antitoxin to BoNT-A; Carr. = carrageenan.

## Data Availability

The original contributions presented in this study are included in this article and Supplementary Material. Further inquiries can be directed to the corresponding authors.

## Supplementary Materials

The following supporting information can be downloaded at https://www.mdpi.com/article/10.3390/toxins17030140/s1, Figure S1: Immunoreactivity of cl-SNAP-25 in BoNT-A-treated animals in ventral horn of the spinal cord de-pends on its transcytosis; Figure S2. Specific neutralization of BoNT-A is not dependent on the antitoxin systemic distribu-tion; Table S1: Baseline paw pressure withdrawal threshold (g) values expressed as mean ± SD; measured before pain induction (carrageenan injection).
