# Supplementary material for: Botulinum Toxin Type A Exerts Direct Trans-Synaptic Action at Bilateral Spinal Nociceptive Circuits
Source: Toxins (Basel). 2025 Mar 14;17(3):140. doi: 10.3390/toxins17030140 (PMC11945969; doi:10.3390/toxins17030140)
Supplement: Supplementary file 1 [file toxins-17-00140-s001.zip › toxins-3448965-supplementary.pdf]

Supplementary Materials: Botulinum Toxin Type A Exerts Direct Trans-Synaptic Action at Bilateral Spinal Nociceptive Circuits

Dalia Nemanić, Petra Šoštarić, Patrik Meglić, Ivica Matak and Lidija Bach-Rojecky

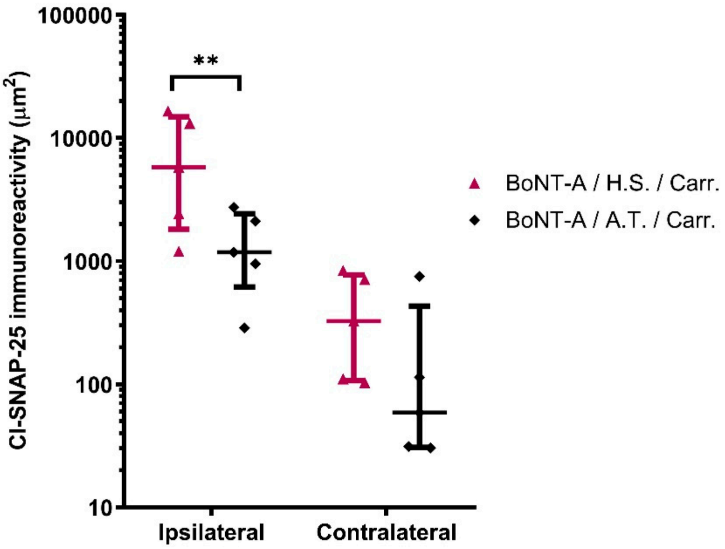

Figure S1. Immunoreactivity of cl-SNAP-25 in BoNT-A-treated animals in ventral horn of the spinal cord depends on its transcytosis. Antitoxin to BoNT-A applied intrathecally reduced the immunoreactivity expression of cl-SNAP-25 in the ipsilateral in ventral horn of spinal cord. Average surface of immunoreactivities of cl-SNAP-25 for each animal was calculated from 3 spinal cord sections per segment (L3, L4, L5/6). Three non-overlapping visual fields per section were analyzed at 40x magnification images (50 μm; surface=436.6μm by 330.2 μm). Number of animals per group = 5. Results are expressed on a logarithmic scale as median with interquartile range; with the statistical analysis performed on square root-transformed data and further analyzed by linear mixed model followed by a two-stage linear step-up procedure of Benjamini, Krieger and Yekutieli to correct for multiple comparisons by controlling the false discovery rate (<0.05)**=p<0.01. Abbreviations: H.S.=horse serum; A.T.=antitoxin to BoNT-A; Carr.=carrageenan.

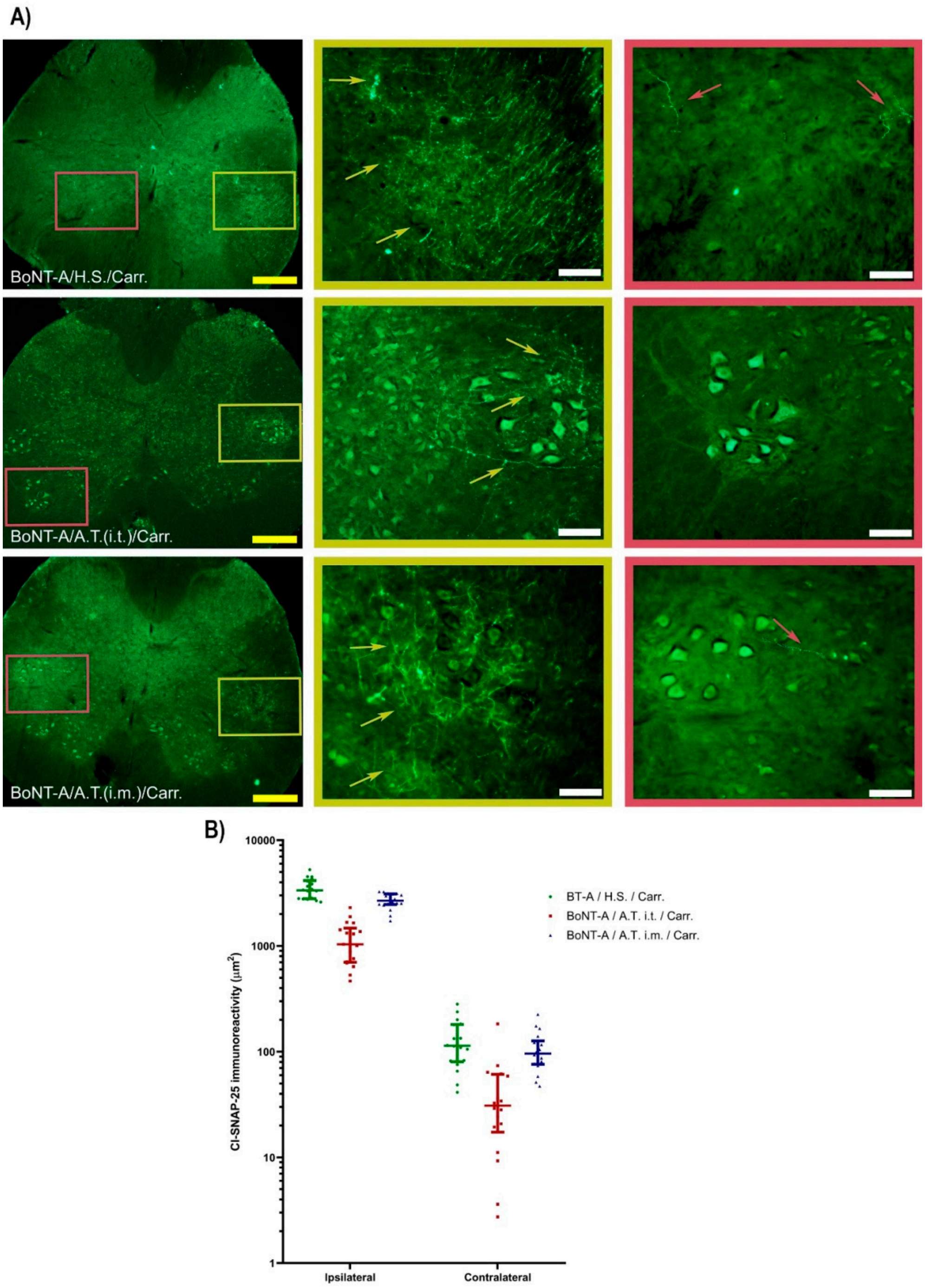

Figure S2. Specific neutralization of BoNT-A is not dependent on the antitoxin systemic distribution. A) Representative images of the cleaved SNAP-25 resulting from BoNT-A proteolytic activity (green immunostaining) in the ipsilateral (yellow frame) and contralateral (red frame) sides of spinal cord ventral horns. Visualization was performed with Axio Observer 7 fluorescent microscope connected to the Axiocam 305 Color camera with 0.63x camera adapter. Scale bar in yellow represents 400 μm (5x magnification) and in white 100 μm (20x magnification). B) Antitoxin to BoNT-A applied intrathecally reduced the immunoreactivity expression of cl-SNAP-25 in the ipsilateral and contralateral side in ventral horn of spinal cord, while antitoxin applied intramuscularly did not significantly change cl-SNAP-25 expression. Average surface of immunoreactivities of cl-SNAP-25 for each animal was calculated from 6 spinal cord sections per L5/6 segment. Three non-overlapping visual fields per section were analyzed at 40x magnification images (50 μm; surface=337.6μm by 281.7 μm). Number of animals per group = 3. Results are expressed as individual values of each spinal cord section for all three animals (in total: 18 values per group). Statistics was not performed due to the low number of animals per group. Abbreviations: H.S.=horse serum; A.T.=antitoxin to BoNT-A; Carr.=carrageenan; i.t.=intrathecally; i.m.=intramuscularly.

Table S1. Baseline paw pressure withdrawal threshold (g) values expressed as mean±SD; measured before pain induction (carrageenan injection).

Treatment group	**Baseline – before BoNT/A and carrageenan treatment**		Baseline – **6 days after BoNT/A treatment; before carrageenan injection**	
	**Ipsilateral paw**	Contralateral paw	Ipsilateral paw	Contralateral paw
F.O./H.S./F.O.	457.8±97.6	473.0±112.4	493.5±113.3	426.6±37.6
F.O./H.S./ Carr.	403.6±81.6	422.8±105.3	471.3±69.1	477.6±53.7
BoNT-A/H.S./Carr.	577.4±156.5	528.4±146.9	492.1±67.1	539.0±92.1
BoNT-A/AT/Carr.	531.3±86.1	488.6±135.4	527.5±86.1	497.5±107.5

Abbreviations: Sal.=saline; H.S.=horse serum; A.T.=antitoxin to BoNT-A; BoNT-A – botulinum toxin A (7U/kg); Carr.=carrageenan.
